# Early detection of reduced left ventricular systolic function by 2D speckle tracking echocardiography in patients with primary mitral regurgitation in a Vietnamese cohort

**DOI:** 10.21542/gcsp.2023.26

**Published:** 2023-09-30

**Authors:** Vu Anh Nguyen, Diem Thi Nguyen, Linh Thi To Ong, An Viet Tran, Bao Lam Thai Tran, Chau Minh Tran

**Affiliations:** 1 Hue University of Medicine and Pharmacy, Hue, Viet Nam; 2 Can Tho University of Medicine and Pharmacy, Can Tho, Viet Nam; 3 Tam Duc Heart Hospital, Ho Chi Minh, Viet Nam

## Abstract

**Background:** Mitral regurgitation (MR) is a common heart valve disease, causing many serious complications in several organ systems, especially the cardiovascular system. The 2D speckle tracking echocardiography (STE) is a new technique for detecting potential cardiac dysfunction when only tissue function abnormalities are present. The study aimed to assess left ventricular (LV) systolic function early by STE in patients with primary MR through global LV deformity along the global longitudinal strain (GLS).

**Methods:** An analytical cross-sectional study was performed on 46 patients with moderate to severe primary MR as recommended by the American Society of Echocardiography (ASE) 2017.

**Results:** The prevalence of patients with GLS reduction with ejection fraction (EF) >60%, New York Heart Association (NYHA) I, and left ventricular internal diameter systolic (LVIDs) <40 mm was 38.1%, 35.7%, and 39.5%, respectively. 100% of patients with EF<60% and LVIDs ≥40 mm had reduced GLS (<16%). The GLS index strongly correlates with the NYHA classification, degree of MR, EF, and echocardiographic parameters.

**Conclusion:** GLS index gives a significant sign in the early detection of cardiac function abnormalities before symptoms or other echocardiographic parameters in patients with MR.

## Introduction

Mitral regurgitation (MR) is a valvular heart disease which has an increasing prevalence with age^1^. MR occurs in 10% of the general population^2^. In particular, left ventricular (LV) dysfunction will progress silently over many years with few or no symptoms. When the symptoms are clear, LV dysfunction may not be reversible, increasing morbidity and mortality rates despite surgery for mitral valve disease^3^. The key issue is determining when to intervene before the LV function reverses. Therefore, early detection of cardiac function abnormalities before symptoms can help clinicians have a better treatment strategy.

Finding reliable echocardiographic parameters for early detection of cardiac function abnormalities before symptoms or changes in classic echocardiographic parameters in patients with MR may help clinicians devise a better treatment strategy, deciding the appropriate timing of surgery to improve postoperative prognosis^3^.

Two commonly used methods to evaluate LV function are M-mode echocardiography and Simpson’s method. However, they only detect cardiac abnormalities when there is a complication of cardiac chamber dilation. Tissue Doppler ultrasonography can also help detect abnormalities of LV function early, but it is angle dependent. Many studies have shown that the echocardiographic markers of myocardial tissue detect potential cardiac dysfunction when there is only abnormal tissue function, no changes in cardiac morphology, and normal ejection fraction (EF). Simultaneously, the echocardiographic technique can assess cardiac function in different directions regardless of angle^4–6^.

In Vietnam, studies using this technique for early detection of cardiac systolic function abnormalities in patients with MR, particularly those without surgical indications, are limited.

## Materials and Methods

### Research objects

An analytical cross-sectional study of 46 patients with moderate to severe primary (organic) MR recommended by the American Society of Echocardiography (ASE) 2017 underwent cardiac Doppler echocardiography at Tam Duc Heart Hospital in Ho Chi Minh City from April 2019 to October 2020. The sample size is calculated with n ≥43. Include Z0.975 = 1.96; *α* = 0.05; *d* = 0.09; *p* = 0.1^2^.

#### Selection criteria

The patient was diagnosed with moderate to severe primary MR according to the recommendations of ASE 2017 by cardiac Doppler in Ho Chi Minh City^7^. The patient consented to participate in the study.

#### Exclusion criteria

Patients with secondary MR, MR due to myocardial infarction, MR combined with severe mitral stenosis and aortic valve stenosis, and heart failure with EF <40% (M-mode or Simpson). The patient had surgery to repair or replace the mitral valve. The patient has a bad ultrasound picture, and tachycardia (determined *via* STE frame rate). The patient did not consent to participate in the study.

### Research content

#### Patient characteristics

General characteristics include (1) age; (2) gender (male and female); (3) causes of MR (mitral valve prolapse (MVP) and rheumatic heart disease).

Clinical characteristics of patients with MR include (1) degree of MR (severe and moderate were assessed by echocardiography and classified according to echocardiographic parameters: In patients diagnosed with mild MR, GLS has begun to decrease and from moderate to severe MR, GLS has already decreased)^7^ (see Appendix A); (2) shortness of breath (including during exertion, during light exercise, and during paroxysmal or lying dyspnea); (3) New York Heart Association (NYHA) stands for classes of heart failure (NYHA I, NYHA II, NYHA III, NYHA IV)^7–9^.

Cardiac Doppler echocardiography to classify primary and secondary MR is assessed through parameters including (1) ejection fraction (EF); (2) end-diastolic volume (EDV); (3) end-systolic volume (ESV); (4) left atrial volume (LAV); (5) pulmonary arterial pressures (PAPs).

Echocardiography parameters indicate that there are images of ischemic or non-ischemic cardiomyopathy with structural abnormalities or motion of the left ventricle, we classify the disease as secondary MR to exclude it.

According to the mitral regurgitation diagnostic criteria, MVP myxomatous changes, degenerative changes, infectious, inflammatory, and congenital causes are the main causes of primary MR. Based on the above causes, secondary mitral MR was excluded from the study^7^ (Appendix B).

#### Characteristics of GLS index and correlation between GLS index and NYHA classification, degree of mitral regurgitation, EF, and echocardiographic parameters.

Characteristics of global longitudinal strain (GLS) index expressed through assessment of LV systolic function by echocardiography of 2D myocardium in patients with moderate to severe primary MR assessed through GLS index through 3 longitudinal sections including (1) Four-chamber view at the apex (4CV); (2) Two-chamber view at the apex (2CV); (3) Three-chamber view at the apex of the heart (3CV).

Determining the correlations between NYHA classification, degree of MR, EF, and echocardiographic parameters with GLS include (1) Correlation between ejection fraction (EF) and GLS; (2) Correlation between the jet area/left atrium (LA) area (%) ratio with GLS; (3) Correlation between vena contracta (VC) and GLS; (4) Correlation between effective regurgitant orifice area (EROA) and GLS; (5) GLS index according to NYHA; (6) Correlation between end-diastolic volume (EDV) with GLS; (7) Correlation between end-systolic volume (ESV) and GLS; (8) Correlation between left atrial volume (LAV) and GLS; (9) Correlation between pulmonary arterial pressures (PAPs) and GLS.

### Early assessment of LV systolic function by left ventricular GLS index in patients with primary MR

GLS decreased <16% with a cutoff value of −19.5% for GLS in the assessment of LV systolic function^6^. When GLS decreased <16%, EF >60%, NYHA I and LVIDs <40 mm all had decreased expression. The prevalence of GLS reduction in patients with MR with NYHA I, EF>60%, and left ventricular internal diameter systolic (LVIDs) <40 mm was determined through 3 parameters including (1) NYHA was divided into 4 classes NYHA I, NYHA II, NYHA III, NYHA IV; (2) LV EF includes 3 ranges of values >60%, 50–60%, <50%; (3) LVIDs include 2 ranges of values <40, ≥40 in patients with moderate and severe primary MR.

#### Data collection

Patients enrolled in the study underwent a data collection process including physical examination and questionnaire, routine M-mode echocardiography, Simpson, mitral flow Doppler, LAV, and deformity analysis of heart muscle.

The STE technique was performed by image acquisition with an average frame rate of 40- 80 fps^10^. To optimize imaging, the area to be examined should be placed at an intermediate depth, and the width of the area should be adjusted just enough. The axial view must pass through the apex or with the short axial view the left ventricular is round, the transverse deformation and torsion results are accurate^11^. Doppler ultrasound is measured in 4CV and 2CV at the apex, with the ultrasound beam aligned with the direction of motion of the area to be examined; preferably, the Doppler angle does not exceed 15°, with the most appropriate speed >100 images/second and at least 4 QRS^11^.

*Data collection tools*: The ultrasonic tissue marking process was performed using an Affiniti 70 ultrasound machine, with a frame rate of 1900 frame/s. QLAB 9.0 software, analyzed by aCMQ software. Probe S4-2, frequency 2–4 MHz.

#### Data analysis

Data analysis was performed using STATA14 software. Quantitative data are expressed as mean ± SD and qualitative data as a percentage. Quantitative data were evaluated by an independent T-test, 1-way ANOVA (if >3 groups were compared) with a normal distribution while qualitative data were evaluated by a chi-squared test. Pearson’s coefficient was used for the correlations. A *p*-value <0.05 was considered to be statistically significant.

#### Ethics Committee Approval

The study was reported and approved by Hue University of Medicine and Pharmacy and the leadership of Tam Duc Heart Hospital, Ho Chi Minh City. (Decision No. 1435 /QÐ-ÐHYD dated 30th July 2014).

## Research diagram

**Figure 1.**
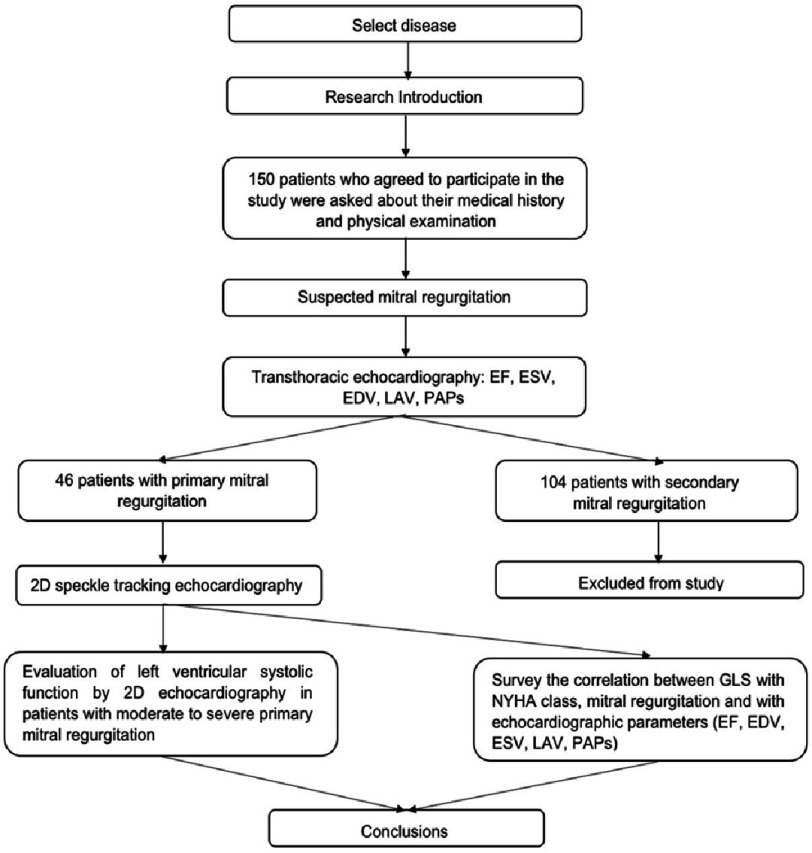
Study flow chart. EF, Ejection fraction; EDV, End-diastolic volume; ESV, End-systolic volume; GLS, Global longitudinal strain; LAV, Left atrial volume; NYHA, New York Heart Association; PAPs, Pulmonary arterial pressures.

The research was carried out according to the flow chart in Figure 1.

**Table 1:** General characteristics and Doppler echocardiographic parameters of the study subjects.

Characteristics	Patients ( *n* = 46) ($\overline{X}+$ SD)
**Gender**	
Male	20 (43.5%)
Female	26 (56.5%)
**Age (year)**	54.2 ± 14.0
**Doppler ultrasound parameters through the chest wall**	
EDV (ml)	100 ± 39
ESV (ml)	33 ± 17
EF%/M mode	69.6 ± 7.5
EF%/Simpson (4CV)	63.9 ± 6.1
LAV (ml)	65 ± 25
PAPs (mmHg)	33 ± 10

**Notes.**

EDV End-diastolic volume ESV End-systolic volume EF Ejection fraction 4CV Four-chamber view LAV left atrial volume PAPs Pulmonary arterial pressures

**Table 2:** Clinical Characteristics and risk factors of study subjects.

Characteristics	Patients ( *n* = 46)
**Causes of MR**	
MVP	40 (87.0%)
Rheumatic heart disease	6 (13.0%)
Total	46 (100%)
**Degree of MR**	
Severe	32 (69.6%)
Moderate	14 (30.4%)
**Sudden onset of Dyspnea**	
During exertion	18 (39.1%)
During light exercise	5 (10.9%)
During paroxysmal or lying dyspnea	3 (6.5%)
**NYHA classification**	
I	28 (60.9%)
II	13 (28.3%)
III	2 (4.4%)
IV	3 (6.5%)

**Notes.**

MR Mitral regurgitation MVP Mitral valve prolapse NYHA New York Heart Association

**Table 3:** Characteristics of ultrasonic parameters.

	Overall ( *n* = 46)	Degree of MR		p
		Severe ( *n* = 32)	Moderate ( *n* = 14)	
	$\overline{X}+$ SD	$\overline{X}+$ SD	$\overline{X}+$ SD	
EF% /M-mode	69.6 ± 7.5	67.7 ± 7.6	74 ± 5.1	<0.01
Simpson (4CV) EF%	63.9 ± 6.1	62.9 ± 6.3	66.4 ± 4.8	>0.05
LAV (ml)	65 ± 25	75.1 ± 21.8	40.3 ± 14.6	<0.01
PAPs (mmHg)	33 ± 10	34.8 ± 10.8	28.9 ± 7.2	>0.05
GLS (-)	16.7 ± 2.9	15.4 ± 2.2	19.7 ± 2.0	<0.01

**Notes.**

EF Ejection fraction 4CV Four-chamber view GLS Global longitudinal strain LAV Left atrial volume MR Mitral regurgitation PAPs Pulmonary arterial pressures

## Results

Table 1 describes the general characteristics and Doppler echocardiographic parameters of the study subjects. We found a predominance of women (56.5%) and an average age of 54.2 ± 14 years. The study recorded the indexes of EDV, ESV, EF%/M mode, EF%/Simpson (4CV), LAV, and PAPS with mean values of 100 ± 39 ml, 33 ± 17 ml, 69.6 ± 7.5 ml, respectively, 63.9 ± 6.1 ml, 65 ± 25 ml, and 33 ± 10 ml.

Table 2 describes the clinical characteristics and risk factors of the study subjects, revealing that 69.6% had severe MR and the remaining 30.4% had moderate MR. The cause of MR due to MVP accounts for 87.0%; the rest is rheumatic heart disease accounting for 13.0%. Subjects had the highest rate of dyspnea during exertion, 39.1%, dyspnea when mild exercise and paroxysmal dyspnea or lying down was 10.9% and 6.5%, respectively. Most of the study subjects had NYHA I heart failure class of 60.9%. The rate of heart failure in classes II, III, and IV, respectively, was 28.3%; 4.4%; and 6.5%.

Table 3 shows that there is a statistically significant difference (p<0.01) in the mean GLS index in the severe MR group of −15.4 ± 2.2% and the moderate MR group is −19.7 ± 2.0%. The echocardiography indicators showed a significant difference between the severe and moderate MR groups.

**Figure 2.**
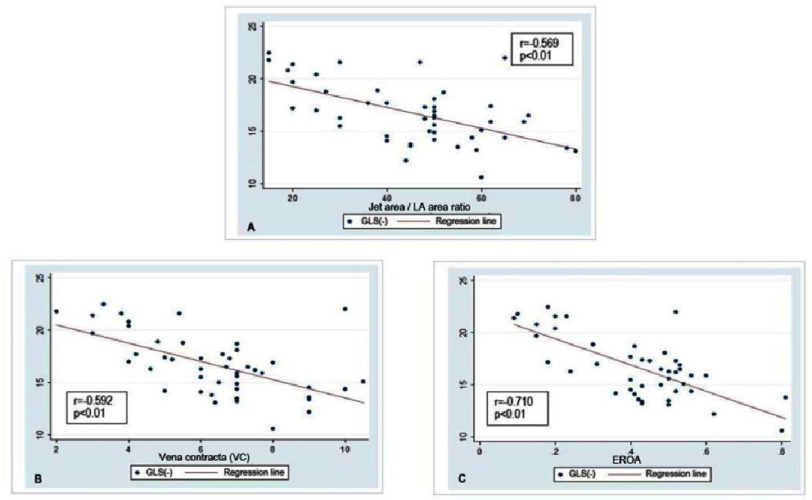
(A–C) Correlation between degree of mitral regurgitation and GLS. EROA, Effective regurgitant orifice area; GLS, Global longitudinal strain.

Analysis of the results (Figure 2), shows a negative correlation between GLS and the jet area/LA area ratio (*r* =−0.569; *p* < 0.01), VC (*r* =−0.592; *p* < 0.01), and EROA (*r* =−0.710; *p* < 0.01). It demonstrates that as the jet area/LA area ratio, VC, and EROA increase, the GLS index decreases. The GLS index indicates that the more severe the MR, the lower the LV systolic function.

**Figure 3.**
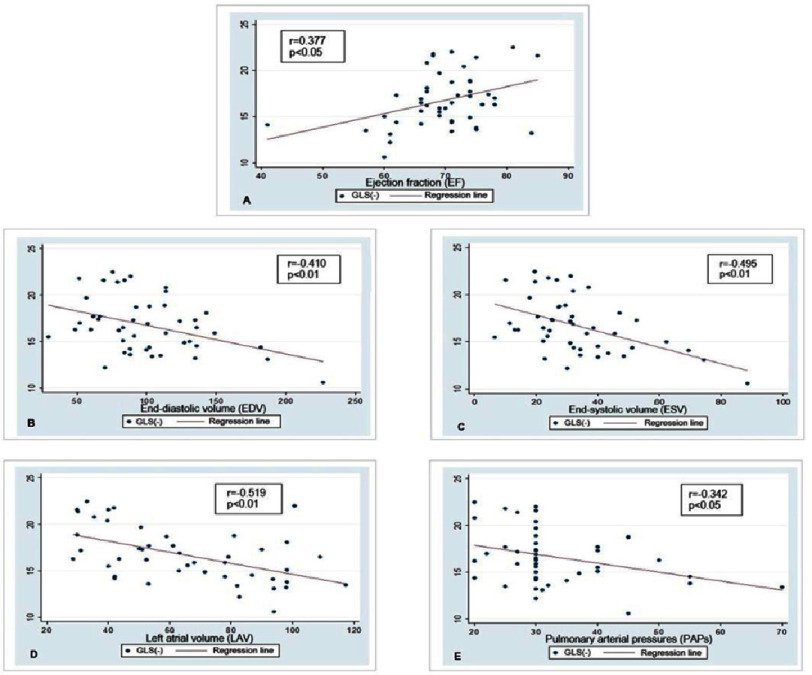
(A–E) Correlation between echocardiographic parameters with GLS. GLS, Global longitudinal strain.

Figure 3 shows that there is a negative correlation between GLS with EDV (*r* =−0.410; *p* < 0.01), ESV (*r* =−0.459; *p* < 0.01), LAV (*r* =−0.519; *p* < 0.01), PAPs (*r* =−0.342, *p* < 0.05). Except that EF has a positive correlation with GLS (*r* = 0.38; *p* < 0.05), the absolute index of GLS increases when the EF index increases.

**Figure 4.**
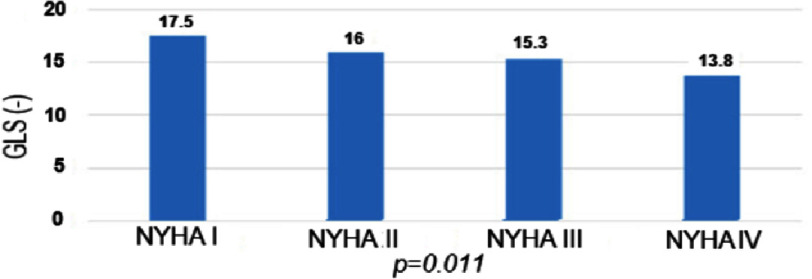
GLS index by NYHA. GLS, Global longitudinal strain; NYHA, New York Heart Association.

The analysis of the relationship between the GLS index according to the NYHA classification (Figure 4) shows a statistically significant difference between the GLS and the NYHA class ( *p* < 0.05). This means that the more severe the NYHA class, the lower the absolute index of GLS.

The results presented in Table 4 show that 35.7% of NYHA I patients had GLS reduction <16%. Patients with NYHA II, III, and IV had a decrease in GLS <16% of 53.9%, 50.0%, and 66.7%, respectively. In patients with EF <50%, EF from 50–60%, 100% of patients with GLS decrease <16%, and EF >60% have 38.1% of patients with GLS decrease <16%. In the group with LV dilatation with LVIDs ≥40 mm, 100% of patients had a decrease in GLS <16%, and in the group with undilated left ventricles LVIDs <40 mm, up to 39.5% of patients had a decrease in GLS <16%.

**Table 4:** Percentage of GLS reduction in patients with NYHA I, EF>60%, and LVIDs<40 mm.

	Overall		GLS reduce <16^*^		Normal GLS ≥16	
	*n*	*%*	*n*	*%*	*n*	*%*
**NYHA**						
I	28	60.9	10	35.7	18	64.3
II	13	28.3	7	53.9	6	46.1
III	2	4.4	1	50.0	1	50.0
IV	3	6.5	2	66.7	1	33.3
**EF%**						
>60%	42	91.3	16	38.1	26	61.9
50–60%	3	6.5	3	100	0	0
<50%	1	2.2	1	100	0	0
**LVIDs**						
<40	43	93.5	17	39.5	26	60.5
≥40	3	6.5	3	100	0	0

**Notes.**

EF Ejection fraction GLS Global longitudinal strain NYHA New York Heart Association LVIDs Left ventricular internal diameter systolic

* GLS decreased <16% with the cut-off value for GLS in assessing the LV systolic function of - 19.5%.

## Discussion

### Patient characteristics

Our study’s gender characteristics showed similar results to the studies of Reckefuss (male 49%, female 51%) and Nguyen Van Tha (male 47.6%, female 52.4%)^12,13^. However, it differs from the Detaint (male 53.57%, female 46.43%), Grigioni (male 67%, female 33%), and Sutton (male 51.02%, female 48.92%) studies^13^.

Our study’s age characteristics are similar to those of other studies such as Rosenhek (55 ± 15), Singh (female 56 ± 13, male 57 ± 11), Sutton (55 ± 19), data cross-sectional surveys, and other studies (median age 59 and 53 years)^13–16^. However, different from some studies such as Reckefuss (mean 42 years), Kobayashi, and Okura (mean 70.9 years)^12,17^.

Because of our study’s smaller sample size compared to the research mentioned above, the age difference between men and women and the prevalence of the disease is not adequately reflected. Nguyen Van Tha’s study differs from ours in terms of MR causes (93.7% MVP and 6.3% rheumatic heart disease) and MR degree (severe MR 50.8% and moderate MR 49.2%) and dyspnea (46% exertional dyspnea and 54% paroxysmal dyspnea at night or while lying down)^11^. Due to the characteristics of the Mekong Delta, people only go to the hospital when they have symptoms, as well as subjective symptoms of the patient.

Our NYHA classification of heart failure differs from some studies such as Nguyen Van Tha (no class I, 46% class II, 39.7% class III, 14.3% class IV), a study of over 150 patients who received mitral valve repair due to mitral valve insufficiency (60% class I, II and 40% class III, IV), and a prospective follow-up study on 551 patients had MR (27.3% class I, 38.7% class II, 34% class III)^18,19^. Because most of the patients in our research sample, which was drawn from a clinic, were asymptomatic or only had minor heart failure symptoms, the rate of heart failure class I and II according to the NYHA is greater than it was for the authors’ study.

### Characteristics of GLS index and correlation between GLS index and NYHA classification, degree of mitral regurgitation, EF, and echocardiographic parameters

In our study, the severe MR group’s GLS indexes tended to decrease statistically significantly (−15.4%) compared to the moderate regurgitation group (−19.7%) ( *p* < 0.001), which showed similarities with the study of Ciro Santoro, Maurizio Galderisi, et al. (2019). In this study, the GLS index in the severe and mild MR group was −17.5% and −20.5%, respectively (*p* < 0.01)^20^. Evaluation of the mean GLS index from our study reveals that it differs from other studies. The Norbert Reckefuss study gave an average GLS of −20.6%^10^. The average GLS in research on MR patients conducted by Ciro Santoro, Maurizio Galderisi, et al. (2019) was −19.8%^21^. The study of N. Bansal, A. Mercadante, et al. (2020) also gave an average GLS of −22.5%^22^. Two other studies (2019) gave GLS indexes of −19.0% and −19.2%, respectively^23,24^. Because normal GLS indexes differ depending on how the site is measured on the myocardium, the type of ultrasound machine used, and the software version used, published results vary significantly. Therefore, because the differences between vendors or software packages are still too great, most documents do not provide a uniformly normal reference value. In addition to software differences, GLS differences are also related to patients (age, gender, etc.) as well as dynamic factors (heart rate etc.).

Some guidelines approve GLS variation within the range of −20% considered normal in healthy individuals. According to Yingchoncharoen et al., LV GLS varied from −22.1% to −15.9%, with an average of −19.7%. Normal GLS according to Philips software and supplier (QLAB 7.1) is −18.9%. Several recent studies have reported lower normal values^25^. Most recently, according to the ACC (2018), in adults, a GLS index <−16% is considered abnormal, and a GLS index >−18% is normal^26^.

Our research discovered an inverse correlation between GLS and VC, jet area/LA area ratio, and EROA, with correlation coefficients of −0.592, −0.569, and −0.710, respectively. According to many reports and literature, the higher the parameters such as VC, jet area/LA area ratio, and EROA, the more severe the MR, so the more severe the MR, the GLS index decreases. Our research found a negative correlation between GLS and VC (*r* =−0.592, *p* = 0.01). This finding is similar to that of Savas Dedeoglu et al., who discovered that in the moderate and severe mitral regurgitation groups, total left ventricular longitudinal strain correlated significantly with the vena contracta (*r* =−0.858; *p* = 0.04), and linear regression results revealed that the only independent association for LV longitudinal strain (model r2 =0.311) was vena contracta (B =−0.858; *p* = 0.04)^27^. Our research differs from Elena Kinova’s study, which found a Pearson correlation between VCW and GLS ( *r* = 0.32, *p* = 0.015) and found that the correlation was moderately positive right^28^. The difference might be explained by the two separate research populations.

The correlation between EF and GLS in our study is similar to that of other studies. Based on GLS, Y. Kobayashi; H. Okura, et al. (2017) discovered a link between EF and LV systolic function^17^. Research S. Yilmaze, R.G. Chelu, et al. (2020) discovered that EF correlates well with GLS^29^. Lima MSM, Villarraga HR, et al. (2017) also showed that the GLS of STE was strongly positively correlated with LVEF, particularly in LV systolic failure^30^. GLS was found to have a significant positive correlation with EF ( *r* = 0.33; *p* < 0.05) in a study that used it to assess the diagnostic accuracy of LV systolic dysfunction by 2D tissue marker ultrasound to predict the severity of coronary artery disease^31^. In patients with preserved EF, reduced GLS (<16%) was associated with a 5.6-fold increased risk of death^32^. Overall, our findings are consistent with those of other authors.

Our study also discovered an inverse correlation between echocardiographic parameters (EDV, ESV, LAV, and PAPs) and GLS, with correlation coefficients of *r* =−0.410, −0.495, −0.519, and −0.342, respectively. This finding is consistent with the findings of Ciro Santoro, Maurizio Galderisi, et al. (2019) on subjects with MR and S. Yilmazer; R.G. Chelu, et al. (2020), who discovered that EDV and ESV were well correlated with GLS (*r* > 0.61, p 0.01)^20,29^.

Our research was similar to other studies that found a statistically significant difference between GLS and NYHA classes (*p* < 0.05). Research by Mizuguchi, et al. also showed a decrease in GLS in hypertensive patients with LV hypertrophy in Asians; this decrease also increased with NYHA class with a statistically significant difference. In Kosmala’s study, the GLS index gradually decreased from NYHA I to IV. The GLS index decreased in patients with heart failure with preserved EF compared to patients with hypertension but no heart failure in Nguyen Thi Diem’s study, and this decline was even greater according to the NYHA class^6^.

### Early assessment of LV systolic function by left ventricular GLS index in patients with primary MR

Our study found that among NYHA I patients, 35.7% had a decrease in GLS <16%; even though the patient had no clinical symptoms, LV systolic function by GLS had already begun to decline. The NYHA classes II, III, and IV decreased the number of patients with GLS by 53.9%, 50.0%, and 66.7%, respectively. Through the study, we found that the prevalence of NYHA class from I to IV decreased, and the GLS decreased by <16% (Especially NYHA I in the asymptomatic group, the GLS decreased early). This difference may suggest that GLS is statistically significant when it has already begun to decrease during the asymptomatic heart failure stage.

In our study, 100% of patients with EF reductions of <50% and from 50%–60% had a GLS decrease of <16%. The findings revealed that in cases with EF at the transition stage of 50–60%, all subjects had reduced GLS. Although EF was only slightly reduced or normal, it was completely abnormal in GLS. In particular, in the group with EF >60%, up to 38.1% of patients had reduced GLS, corresponding to the compensated MR stage, but LV systolic function through the GLS index began to decrease. This demonstrates that GLS has a higher sensitivity than EF in detecting LV contractility abnormalities early.

According to Chin-in Lo et al., EF is insufficient to assess potential LV dysfunction in patients with preserved EF. In this case, GLS will superior EF in detecting early cardiac dysfunction in this group of patients^6,33^.

Ciro Santoro, Maurizio Galderisi, et al. (2019) found a correlation between GLS and EF and other echocardiographic parameters in 504 patients with mild to severe MR, which was similar to our findings. In particular, GLS was shown to be superior to EF in detecting LV systolic dysfunction in MR patients^20^. Our findings in normal patients show that even when EF has not yet decreased, GLS has already begun to decrease. This finding suggests that GLS has an early prognostic value in detecting LV dysfunction. To further demonstrate GLS’s superiority, there is compelling evidence that LV-GLS has a higher predictive value than LVEF in predicting LV dysfunction and serious cardiac events in many cardiovascular diseases^34^.

Similarly, in the LV dilation group with LVIDs ≥40 mm, 100% of subjects had decreased GLS, while in the undilated left ventricle group LVIDs <40 mm, 39.5% of subjects had also decreased GLS.

## Limitations and implementations

Our study achieved many positive results, but it has some limitations. That is the sample size is small, and our research was conducted at only one center. To better understand the significance of this method in MR subjects, the sample size, and scope of the study must be expanded. More longitudinal studies with larger sample sizes or multicenter studies with longer follow-ups are required to assess the risk of heart failure between groups that have and do not have GLS changes on STE.

This study demonstrated that STE is superior to other ultrasound methods and that the GLS index on STE can be used to guide management, stratify prognosis, and determine an appropriate time for surgical intervention in patients with mitral regurgitation. Furthermore, in a large number of asymptomatic patients with severe primary mitral regurgitation and preserved ejection fraction, resting GLS may be shown to be a strong predictor of long-term prognosis.

However, assessing the group’s decline in GLS on STE following surgical intervention is beyond the scope of the study. Therefore, further studies are needed to investigate the correlation of GLS decline on STE in the group before and after surgical intervention for primary mitral regurgitation.

## Conclusions

Research results have shown that 2D STE helps in the early detection of abnormal manifestations of LV systolic function of the heart in patients with primary MR. In particular, for patients with no indication for surgery, the STE technique proved more effective than conventional ultrasound methods. The accuracy of diagnosing LV systolic dysfunction by 2D STE will increase further when combined with the LV GLS index.

## Author statements

Conception: Vu A. Nguyen.

Design: Linh T. T. Ong.

Supervision: Diem T. Nguyen.

Funding: Diem T. Nguyen.

Materials: An V. Tran.

Data collection and/or processing: Linh T. T. Ong, Bao L. T. Tran, and Chau M. Tran.

Analysis and/or interpretation: An V. Tran.

Literature review: Diem T. Nguyen.

Writer: Bao L. T. Tran and Chau M. Tran.

Critical review: Vu A. Nguyen.

## Appendix B. Diagnosis of mitral regurgitation^7^

**Table:**

**Primary MR (leaflet abnormality)**	
- MVP myxomatous changes	- Prolapse, flail, ruptured or elongated chordae
- Degenerative changes	- Calcification, thickening
- Infectious	- Endocarditis vegetations, perforations, aneurysm
- Inflammatory	- Rheumatic, collagen vascular disease, radiation, drugs
- Congenital	- Cleft leaflet, parachute MV
**Secondary MR (ventricular remodeling)**	
- Ischemic etiology	- Artery disease
- Nonischemic cardiomyopathy: Annular dilation	- Atrial fibrillation, restrictive cardiomyopathy
